# Eat like an athlete: insights of sports nutrition science to support active aging in healthy older adults

**DOI:** 10.1007/s11357-021-00419-w

**Published:** 2021-07-20

**Authors:** Sara Y. Oikawa, Tristin D. Brisbois, Luc J. C. van Loon, Ian Rollo

**Affiliations:** 1 Gatorade Sports Science Institute, PepsiCo Life Sciences, Global R&D, 5500 34th Street West, Bradenton, FL 34210 USA; 2 PepsiCo Life Sciences, Global R&D, Purchase, NY USA; 3 Department of Human Biology, NUTRIM School for Nutrition, Toxicology and Metabolism, Maastricht University, Maastricht, Netherlands; 4 School of Sports Exercise and Health Sciences, Loughborough University, Loughborough, UK

**Keywords:** Protein, Creatine, n-3PUFA, Carbohydrate periodization, Skeletal muscle

## Abstract

Skeletal muscle mass losses with age are associated with negative health consequences, including an increased risk of developing metabolic disease and the loss of independence. Athletes adopt numerous nutritional strategies to maximize the benefits of exercise training and enhance recovery in pursuit of improving skeletal muscle quality, mass, or function. Importantly, many of the principles applied to enhance skeletal muscle health in athletes may be applicable to support active aging and prevent sarcopenia in the healthy (non-clinical) aging population. Here, we discuss the anabolic properties of protein supplementation in addition to ingredients that may enhance the anabolic effects of protein (e.g. omega 3 s, creatine, inorganic nitrate) in older persons. We conclude that nutritional strategies used in pursuit of performance enhancement in athletes are often applicable to improve skeletal muscle health in the healthy older population when implemented as part of a healthy active lifestyle. Further research is required to elucidate the mechanisms by which these nutrients may induce favourable changes in skeletal muscle and to determine the appropriate dosing and timing of nutrient intakes to support active aging.

## Introduction

Population aging is a global phenomenon. It is estimated that 1 in every 6 individuals will be over the age of 65 by the year 2050, an increase of 45% from 2019 [1]. Aging is associated with the loss of skeletal muscle mass and strength, termed sarcopenia. Sarcopenia is measurable within the 5th decade of life and is associated with loss of muscle mass at a rate of ~ 1% per year [2], loss of muscle strength (e.g. 1-repetition maximum) at ~ 3% per year [3], and loss of muscle power (e.g. force and speed of movement) at ~ 8% per year [4]. Importantly, these decrements in muscle function are associated with the loss of independence, a decline in the ability to perform activities of daily living [5], and are linked with several negative metabolic health outcomes [6]. Thus, strategies to augment or maintain skeletal muscle mass and its functional capacity are a primary consideration in preserving the quality of life of aging adults.

The enhancement of skeletal muscle mass and function are also primary objectives for many athletes. In this population, specific exercise and nutrition regimens are utilized to optimize skeletal muscle remodelling and often to stimulate muscle hypertrophy. Despite athletic intervention strategies being performance focused, many principles are directly applicable to skeletal muscle health in older adults. Both athletic and older populations can accumulate training hours to achieve competitive goals [7]. However, for the general healthy older population, physical activity provides benefits beyond athletic achievements, such as maintaining independence, reducing the risk of falls, and establishing/continuing social interaction [8]. Consequently, the nutrition intervention strategies discussed in this review should be applied in the context of supporting the completion of, and maximizing the benefits of, physical activity and, as such, to support more active aging.

To this end, this review will discuss sports nutrition strategies used by athletes and discuss their potential application to the healthy aging population with a focus on skeletal muscle mass and function. For the purpose of this review, healthy older adults are classified as those people free of health conditions or medications that may impact clinical outcomes of the nutrition interventions.

## Dietary protein

Recommendations for daily protein intakes for athletes (1.2–2.0 g/kg/day) [9] are above the Institute of Medicine guidelines for adults (> 18 years) (0.8 g/kg/day) [10]. Athletes require a greater dietary protein intake to support the repair and replacement of damaged proteins, muscle hypertrophy (if desired), and the reconditioning of various tissues (muscle, bone, and connective tissues) following training [10]. Further, a recent meta-analysis showed that protein supplementation accounted for 9% of total strength gained during resistance exercise (RE) training [11]. A previous meta-analysis [12] has also highlighted the benefits of increasing daily protein intake on lean body mass (LBM) during training in both younger and older adults. Protein supplementation may also facilitate adaptation to endurance exercise training. Greater improvements in V̇O_2_max were observed following 12 weeks of cycling in a group ingesting additional casein protein compared to a placebo group [13].

Both aerobic and RE have been shown to increase muscle protein synthesis (MPS) in older people [14]. This may form the basis of the observation that participation in life-long physical activity attenuates the loss of LBM with age [15]. Furthermore, participation in regular physical activity results in better outcomes of physical function (strength, gait speed) compared to sedentary older people [16]. Importantly, exercise and protein ingestion act independently to stimulate MPS [17]. However, when protein is consumed in close temporal proximity to exercise, they act synergistically to increase MPS greater than either stimulus independently [17]. Thus, combining exercise with protein feedings represents a potent strategy to enhance anabolism in both young [18] and older people [19].

Indeed, long-term RE can augment anabolic sensitivity and augment skeletal muscle mass and strength in older adults [20]. However, older people experience a decline in the skeletal muscle protein synthetic response to protein ingestion, termed anabolic resistance [21] and, therefore, nutritional strategies to optimize the benefits of physical activity are imperative. Of note, ingesting 20 g of whey protein (the quantity recommended to enhance MPS in most athletes [22]) does not maximize MPS in older people either acutely [19] or chronically when combined with RE [23]. These findings are supported by data suggesting that older people require ~ 40% more dietary protein after exercise to maximize MPS, compared to that required by younger adults [21]. For example, a serving of 35 g (~ 0.4 g/kg/serving) of protein is required to maximize rates of MPS for the average US male over 60 years (88 kg) [24].

**Fig. 1:**
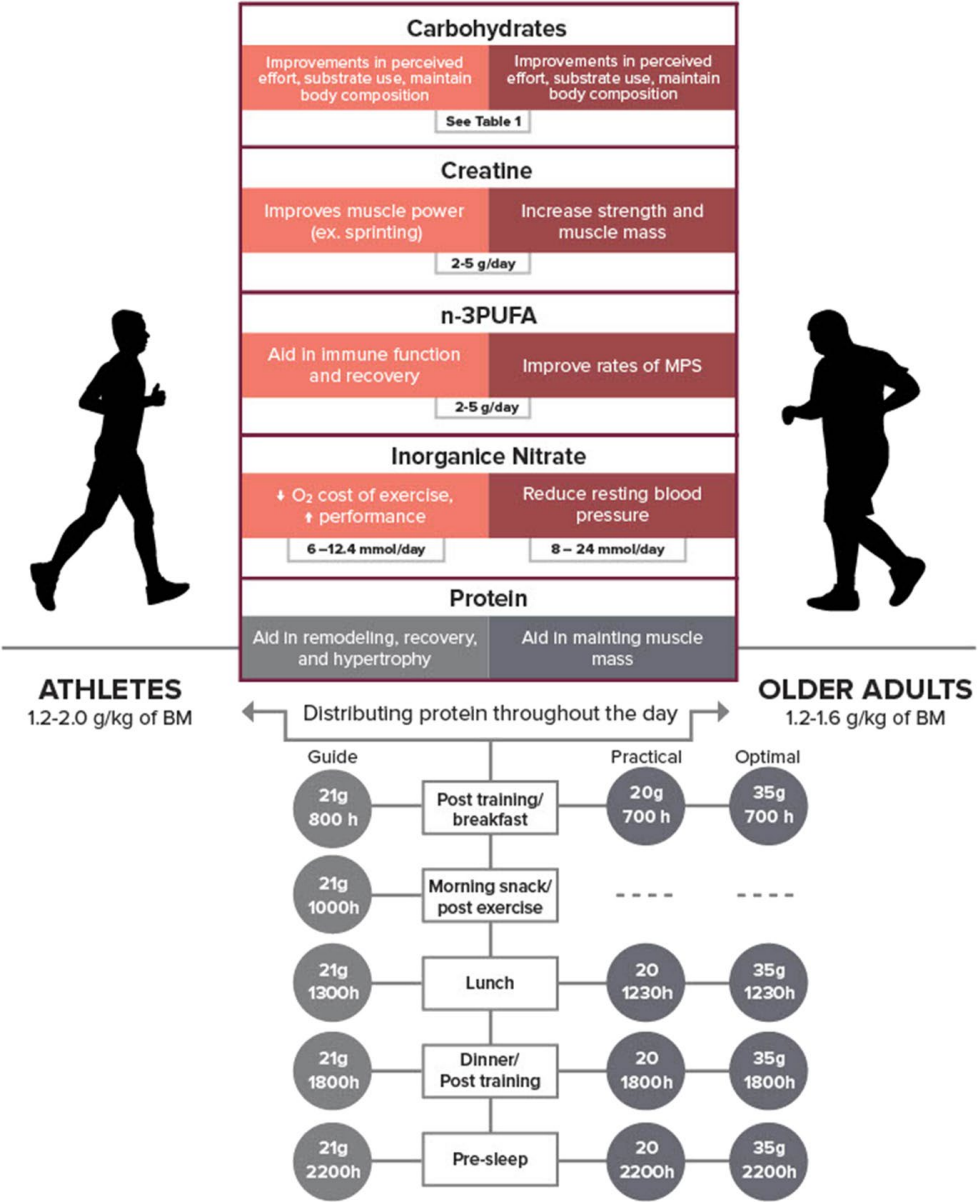
Impact of protein, creatine, omega-3 s (n-3PUFAs), inorganic nitrate/beetroot juice, and carbohydrate periodization on skeletal muscle in athletes (pink) and older people (red). Proposed daily protein consumption layout and dosage for both athletes (light) and older people (dark). For older people, we present both a practical protein dose (similar to athletes) and an optimal protein dose. Optimal protein intake following exercise is body mass dependent and can be calculated by 0.24–0.3 g/kg of body mass for young people (< 30 years of age), and 0.4 g/kg of body mass for adults (> 60 years of age) [21]. BM, body mass

Nonetheless, we acknowledge that ~ 35 g of protein can be a daunting quantity of protein for older people to ingest at a single meal. Despite a lower protein target than “optimal”, aiming to consume protein quantities similar to athlete recommendations (~ 20 g protein/serving) may be considered a more practical approach. Furthermore, 20 g protein per eating occasion will also contribute to achieving augmented daily protein recommendations for older people (1.2–1.6 g/kg/day) [25]. To this end, a daily timetable of protein ingestion for both athletes and older people is displayed in Fig. 1 with examples of protein-containing meals at the suggested doses in Table 1. Spacing protein ingestion more evenly throughout the day (3–4 doses or meals, 0.25 g/kg and 0.4 g/kg of protein in young and older people, respectively) is also considered optimal to maximize the benefits of daily protein ingestion and benefits from exercise-induced anabolic sensitivity [26]. Typically, adults in both the USA [27] and the UK [28] exhibit a highly skewed pattern of daily protein intake where more than 50% of total protein is consumed at one meal (lunch or dinner). When examined in free-living older people, ingesting higher amounts of total daily protein more evenly distributed throughout daily meals resulted in greater LBM and appendicular lean mass [29] which may be explained by achieving increases in rates of MPS multiple times per day (rather than once per day if protein is consumed in a skewed manner).

**Table 1:** Examples of protein sources for athletes (or practical meals for older adults) at ~ 20 g, and optimal meals for older adults at ~ 35 g at each eating occasion

	Guide/practical ~ 21 g pro/meal	Optimal (> 60 years) ~ 35 g pro/meal
Breakfast/post-training	1 cup cooked oatmeal (6 g protein) 1/2 scoop whey protein isolate (15 g protein)	2 eggs (12 g protein) 2 slices whole grain bread (7 g protein) ½ cup low fat cottage cheese (14 g protein)
Morning snack	1/2 cup low fat cottage cheese (14 g protein) 10 roasted almonds (6 g protein)	1 scoop whey protein isolate (30 g protein) ¼ cup Greek yogurt (5 g protein)
Lunch	100 g turkey breast (15 g protein) 2 slices whole grain bread (7 g protein)	100 g turkey breast (15 g protein) 2 slices whole grain bread (7 g protein) 1.5 cups 1% milk (13 g protein)
Dinner/post-training	3 oz chicken breast (24 g protein)	4 oz chicken breast (32 g protein) ½ cup cooked quinoa (3.5 g protein)
Pre-sleep	20 g casein protein shake	35 g casein protein shake

Importantly, the synergistic interaction of exercise and nutrition is not unique to protein. Similar dietary strategies can be and have been used with various nutrients and nutritional supplements (creatine, omega 3 s, dietary nitrate) to augment LBM and strength to enhance physical performance in athletes. The following sections will discuss nutrients or supplements used by athletes in conjunction with exercise training to improve skeletal muscle performance and how they may also favourably impact skeletal muscle health in older people. These findings have been summarized in Fig. 1.

### Creatine

To optimize or maintain elite performance, athletes participate in RE training in order to increase or sustain skeletal muscle mass and strength. Creatine monohydrate is a popular dietary supplement amongst athletes due to its ergogenic effects and its ability to enhance the benefits of exercise on skeletal muscle [30]. Oral creatine monohydrate supplementation can improve high intensity exercise capacity by increasing skeletal muscle stores of phosphocreatine (PCr) by 20–40% [31]. The heightened ability to train at higher workloads has been shown to contribute to increases in muscle mass and strength when combined with exercise training [32]. Supplemental creatine is ergogenic for athletes requiring quick bursts of power or strength (e.g. weight lifting) or for athletes whose sports performance requires intermittent sprinting [33] and can improve the performance of high intensity exercise by 10–20% [30].

In older people, higher concentrations of muscle PCr are associated with greater muscle volume and greater peak knee extensor power [34] while, conversely, sarcopenic older adults exhibit a lower muscle PCr content compared to non-sarcopenic controls [34]. Older adults exhibit greater increases in LBM [35, 36], muscle thickness [37], and strength [35, 36] during resistance training (3 × per week) [38] when supplementing with creatine (3–5 g/day). However, it should be noted that all older adults in the aforementioned studies were considered healthy and free of chronic conditions which may impact the study findings. Indeed, a meta-analysis of older participants (50–72 years) reported a weighted mean difference of 1.37 kg and 0.24 kg for LBM and leg press 1-repetition maximum respectively, in individuals supplemented with creatine [38]. Importantly, in over 700 participants, there were no reported adverse events related to kidney or liver function following prolonged creatine supplementation [38]. Interestingly, muscle stem cells (satellite cells) also appear to respond to creatine supplementation through the upregulated expression of their regulatory factor MRF-4 [39]. Both satellite cell activity and the satellite cell pool are reduced with increasing age [40], and therefore, mechanisms of action linked with beneficial effects of creatine monohydrate ingestion may extend beyond the muscle’s capacity for enhancements in exercise capacity in older people.

### Omega-3/n-3 polyunsaturated fatty acids (n-3 PUFA)

Athletes ingest omega-3 supplements in pursuit of a wide variety of reported performance and health benefits including reduced inflammation, improved recovery (following injury), and enhanced immunity, and in some cases, in an effort to increase skeletal muscle metabolic efficiency [41, 42]. Dietary supplementation with omega-3 fatty acids (n-3PUFA) has been shown to enhance performance through many of the pathways described above; however, of particular importance to skeletal muscle is the potential for n-3PUFAs to sensitize skeletal muscle to the anabolic effects of protein ingestion through alterations to the skeletal muscle phospholipid membrane [43]. The increase in polyunsaturated fatty acids into the skeletal muscle membrane improves the transport of nutrients, such as amino acids, into muscle, increasing rates of MPS [44]. Indeed, in healthy young adults, ingestion of 4 g/day of n-3PUFAs for 8 weeks improved the MPS response to amino acid administration in comparison to a placebo, suggesting that n-3PUFAs may enhance amino acid transport [45]. An additional benefit to athletes is the finding that n-3PUFAs may reduce muscle soreness following exercise. In competitive soccer players, muscle soreness was significantly lower during the 72 h post-exercise recovery period in the group consuming 1.1 g of n-3PUFAs combined with protein and carbohydrates compared to ingesting protein and carbohydrate alone following 6 weeks [46]. This improvement was attributed to a reduction in muscle damage, with a 39% reduction in creatine kinase in the n-3PUFA group [46].

In healthy older people (65–80 years), prolonged supplementation with n-3PUFAs has been shown to enhance rates of MPS and therefore may aid in the retention of, or facilitate increases in, muscle mass with age [47]. Prolonged ingestion (6 months) of 4 g of n-3PUFAs was associated with increased thigh muscle volume (3.6%), hand-grip strength (2.3 kg), and 1-RM muscle strength (4.0%) in comparison to placebo (4 g/day corn oil) [47]. These improvements were attributed to improved amino acid transport into the intracellular pool, following protein feeding [45].

Of note are the potential sex-based differences in the efficacy of n-3PUFAs to improve skeletal muscle anabolism. The completion of 18 weeks of RE training in combination with 3 g/day of n-3PUFA significantly increased isometric strength and muscle quality (torque/cross sectional area) in older women, with no differences between treatment groups in older males [48]. These findings support previous studies that have shown no benefits of supplementing 3 g/day of n-3PUFA in older men completing a 12-week resistance training intervention on measures of LBM, strength, or functional outcomes [49], whereas a study in older women providing 2 g/day of n-3PUFAs for 150 days increased peak torque, rate of torque development, chair stand performance, and muscle activation to a greater extent than the control group [50]. Thus, older females may benefit to a greater extent from supplementation with n-3PUFAs at doses ≥ 2 g/day. Future research should aim to provide insight into the mechanisms driving favourable skeletal muscle health outcomes and sex-based differences with n-3PUFA supplementation in older people, as well as optimal timing and dosages. Additionally, future research involving individuals with chronic health condition should be considered in an effort to generalize any insights on the advantages of n-3PUFA to a more diverse population of older adults.

## Inorganic nitrate (NO_3_^−^)

Enhanced vascular function benefits all physiological systems, in part through the heightened ability to transport nutrients to tissues. Athletes often ingest dietary sources of inorganic nitrate (NO_3_) (e.g. beetroot juice) due to its potential impact on aerobic exercise performance. Though there is debate as to whether elite athletes can benefit from beetroot/inorganic nitrate, supplementation has been shown to benefit recreationally, and moderately trained individuals [51]. Consumption of NO_3_ is thought to improve exercise performance through the restoration of inorganic nitrate cycling resulting in an augmented vasodilatory response above exercise alone [51]. Indeed, the acute supplementation with 0.1 mg/kg of sodium nitrate reduced oxygen cost during cycling while also improving oxidative phosphorylation in young men [52]. Similarly, 6 days of beetroot juice supplementation (11.2 mM of nitrate/day) resulted in a reduced oxygen cost of exercise and an increased time to task failure during intense exercise in moderately trained men [53]. Importantly, although augmented vascular function is effective and beneficial for performance, the benefits of enhancing vascular function extend well beyond the scope of sport.

Of particular interest is the finding that diets rich in inorganic nitrate are associated with lower blood pressure due to improvements in endothelial mediated vasodilation and increased nitric oxide availability [54]. Increasing age is associated with arterial stiffening, impaired vasodilation, and endothelial dysfunction wherein the nitric oxide pathway responsible for maintaining vascular homeostasis is disrupted [55]. Importantly, supplementation with dietary nitrate or diets high in NO_3_ appear to more efficacious than isolate sources of inorganic nitrate [56]. NO_3_ ingestion has been successful as both a lone intervention and complimentary therapy to improve vasodilation and lower blood pressure in older adults [57]. Recent meta-analyses have reported that NO_3_ supplementation improves indices of vascular function in older people [57, 58] where NO_3_ ingestion between 316 and 860 mg/day resulted in a decrease in systolic and diastolic blood pressure by 3.55 and 1.32 mmHg, respectively [57]. Importantly, increasing vasodilatory capacity may result in increased nutrient delivery, specifically of amino acids, to skeletal muscle, augmenting anabolic sensitivity and potentially aiding in the maintenance of skeletal muscle mass over time [59]. To date, one study has examined the effects of dietary NO_3_ in combination with a protein or amino acid supplement to determine elucidate any synergistic effect on aging skeletal muscle. Kouw et al. examined the acute effects of NO_3_ provision in combination with a meal-like amount of protein (20 g) in older adults with type 2 diabetes and found that NO_3_ supplementation did not augment post-prandial MPS [60]. However, no study has examined the effects of chronic NO_3_ and protein supplementation and the impact on skeletal muscle health in older adults. Nevertheless, increasing dietary intake of inorganic nitrate presents a promising strategy to improve vascular health and skeletal muscle health in older people.

## Carbohydrate periodization

Carbohydrate periodization refers to adjusting the daily intake of carbohydrate and carbohydrate intake during exercise to match the demands and objectives of athletic training [61]. Athletes ingest carbohydrate during exercise competition in pursuit of performance benefits [62, 63]. There are multiple mechanisms by which carbohydrate intake may positively influence performance, from improved perception of effort, to preserving a supply of substrate for muscle contraction [64]. However, during daily training activities, athletes modify their carbohydrate intake depending on the duration and intensity of the exercise that is performed. This approach is utilized by athletes to match their training objectives and for body composition management.

For older people, physical activity is the primary driver to benefit health-related outcomes, as well to maintain physical capabilities. Although performance is not a primary objective for the general healthy aging population, ingesting carbohydrate during exercise may improve the overall experience [65]. Increased persistence during physical activity and increasing the adherence to an exercise regimen are key considerations in promoting exercise. Equally, on rest days or days of no structured physical activity, the main determinant of energy expenditure is fat-free mass. Failing to match the declining energy needs with appropriate decline in energy intake will accumulatively lead to increased body fat content. Increased body fatness and increased abdominal obesity are linked to the increased incidence of non-insulin-dependent diabetes mellitus amongst older people [66]. Thus, guidance of how carbohydrate intake may be modified to match daily energy requirements of exercise is presented in Table 2.

**Table 2:** Daily carbohydrate intake guidelines based on physical activity level. Suggested ranges accommodate likely variations in individual goals specifically related to body composition. Lower intakes should be targeted to accommodate weight loss and fat loss, whereas upper targets should be considered for weight maintenance or gain

	Daily physical activity level	Activity duration (min)	Example activity	Target carbohydrate intake g/kg BM/day
Rest day	Light	0	Daily living	3–5
Exercise (3–5 days/week)	Moderate	20–60	Brisk walking, swimming, cycling, general yard work, gardening, Yoga, Resistance exercise*, Yoga, calisthenics	5–6
	Heavy	60 +	Jogging, running, swimming laps, heavy yard/gardening work	5–8

## Future directions

Given the growing population of older people across the globe, there is an increased need for the determination of effective nutritional strategies to offset the development of age-related conditions (sarcopenia, osteoporosis, dementia, etc.). Though the determination of optimal dosing and timing strategies specific to older people are still to be elucidated, the potential to combine supplemental protein with the aforementioned ingredients to enhance muscle protein anabolism is promising and can be undertaken with little concern for harm. To date, few studies have studied the chronic effects of protein supplementation in combination with creatine, n-3PUFAs, and/or inorganic nitrate. Given the substantial heterogeneity in responses to nutrition and exercise in both young and older people [67], future research should examine chronic ingestion of these nutrients in combination and their impact on muscle health.

It is acknowledged that physiological factors associated with aging, such as insulin resistance, incidence of chronic inflammation, and interactions with medications, will likely result in differential impacts of nutritional supplementation on skeletal muscle health. These differences may be augmented when comparing clinical aging populations to healthy peers or indeed to healthy young athlete populations. Thus, future research investigating the impact of nutrient and exercise interactions should include a robust diversity of older adults with varying health status.

Importantly, many factors impact the rate of sarcopenia and dynapenia in aging adults. Stimuli such as physical inactivity and presence of chronic conditions exert greater influence on the loss of muscle mass and strength than alterations in nutrition. Nevertheless, optimizing nutrition that may augment or attenuate the decline in skeletal muscle health with aging, particularly when combined with exercise, should be considered when tailoring nutritional strategies to older people.

Lastly, although this review focuses on the benefits of individual nutrient supplementation to improve the skeletal muscle health of older adults, consuming nutrients such as protein, creatine, n-3PUFAs, and inorganic nitrate, through whole food sources (ex. beetroot, rocket, spinach) may be of a significant benefit to older people [68]. Indeed, food matrices, which dictate, in part, how food components are structured and interact, may significantly influence the anabolic properties of a nutrient [69]. For example, exercising young men consuming protein from a whole egg in comparison to an isonitrogenous amount of egg whites experienced greater increases in MPS [70]. These effect were independent of total leucine availability [70]. However, reductions in appetite [71] and difficulties with mastication [72] may reduce the ability of older people to consume adequate levels of anabolic nutrients through food sources. As such, dietary supplementation of individual nutrients may be considered.

As mentioned, the maintenance of skeletal muscle mass and strength is imperative to sustain mobility and the ability to carry out activities of daily living in older people [73]. Similarly, the maintenance of cognitive function or the attenuation of cognitive decline is a crucial requirement for independent living [74]. Dietary factors are also strong modulators of cognitive function in aging, wherein high consumption of antioxidants and poly- and mono-unsaturated fats can induce positive effects on cognitive health outcomes in older adults [75]. The ingredients revised as part of this review were selected based on their efficacy to enhance skeletal muscle anabolism in athletes and older people. However, the ingestion of the dietary compounds discussed in this review is not exclusive to the targeted tissues but available to the systemic circulation. Importantly, each ingredient has shown some benefit for cognitive health [76–78] albeit in a limited capacity. As such, future research should aim to determine the impact and benefits of these nutrients both on skeletal muscle but also to brain health in aging populations.

## Summary

In summary, nutritional strategies used by athletes in pursuit of performance enhancements are applicable to improve skeletal muscle health in healthy older people. Importantly, nutritional interventions such as those discussed as part of this review are not sufficient to counteract sarcopenia alone. Along with dietary interventions, increasing physical activity and reducing sedentary behaviour are of significant importance in the pursuit of healthy aging. More research is required to elucidate the mechanisms by which various nutrients may induce favourable changes in skeletal muscle and determine the quantities and timing of nutrient intake to support active aging.

## Data Availability

Not applicable.

## Abbreviations

NO_3_: Inorganic nitrate
LBM: Lean body mass
MPS: Muscle protein synthesis
n-3PUFA: N-3 polyunsaturated fatty acid
PCr: Phosphocreatine
RE: Resistance exercise
